# Comparative transcriptome and co-expression network analysis revealed the genes associated with senescence and polygalacturonase activity involved in pod shattering of rapeseed

**DOI:** 10.1186/s13068-023-02275-6

**Published:** 2023-02-07

**Authors:** Umer Mahmood, Xiaodong Li, Mingchao Qian, Yonghai Fan, Mengna Yu, Shengting Li, Ali Shahzad, Cunmin Qu, Jiana Li, Liezhao Liu, Kun Lu

**Affiliations:** 1 College of Agronomy and Biotechnology, Southwest University, Chongqing, 400715 China; 2 Academy of Agricultural Sciences, Southwest University, Chongqing, 400715 China; 3 Engineering Research Center of South Upland Agriculture, Ministry of Education, Chongqing, 400715 China

**Keywords:** Rapeseed, Pod shattering, Senescence, PG activity, Co-expression network

## Abstract

**Background:**

The pod shattering (PS) trait negatively affects the crop yield in rapeseed especially under dry conditions. To better understand the trait and cultivate higher resistance varieties, it’s necessary to identify key genes and unravel the PS mechanism thoroughly.

**Results:**

In this study, we conducted a comparative transcriptome analysis between two materials significantly different in silique shatter resistance lignin deposition and polygalacturonase (PG) activity. Here, we identified 10,973 differentially expressed genes at six pod developmental stages. We found that the late pod development stages might be crucial in preparing the pods for upcoming shattering events. GO enrichment results from *K*-means clustering and weighed gene correlation network analysis (WGCNA) both revealed senescence-associated genes play an important role in PS. Two hub genes *Bna.A05ABI5* and *Bna.C03ERF/AP2-3* were selected from the MEyellow module, which possibly regulate the PS through senescence-related mechanisms. Further investigation found that senescence-associated transcription factor *Bna.A05ABI5* upregulated the expression of *SAG2* and *ERF/AP2* to control the shattering process. In addition, the upregulation of *Bna.C03ERF/AP2-3* is possibly involved in the transcription of downstream *SHP1/2* and LEA proteins to trigger the shattering mechanism. We also analyzed the PS marker genes and found *Bna.C07SHP1/*2 and *Bna.PG1/2* were significantly upregulated in susceptible accession. Furthermore, the role of auxin transport by *Bna.WAG2* was also observed, which could reduce the PG activity to enhance the PS resistance through the cell wall loosening process.

**Conclusion:**

Based on comparative transcriptome evaluation, this study delivers insights into the regulatory mechanism primarily underlying the variation of PS in rapeseed. Taken together, these results provide a better understanding to increase the yield of rapeseed by reducing the PS through better engineered crops.

**Supplementary Information:**

The online version contains supplementary material available at 10.1186/s13068-023-02275-6.

## Background

Rapeseed (*Brassica napus*, 2*n* = 38, AACC) is an important allopolyploid oil crop that belongs to the Brassicaceae family and developed from the interspecific cross between *Brassica oleracea* (2*n* = 18, CC) and *Brassica rapa* (2*n* = 20, AA) [1]. Rapeseed yield has always been a critical factor, as asynchronous flowering causes PS upon maturity particularly when the crop is harvested after it has fully ripened. The precise harvesting time is a great challenge for growers due to the intermittent seed maturity; it generally accounts for 8–10% yield losses but can exceed up to 50% under severe environments [2, 3]. To overcome the yield losses, early harvesting yields low-quality oil due to chlorophyll contamination [4]. However, the PS mechanism is still ambiguous and needs to be explored at a broader level to decrease the yield losses. This trait must be considered critical for future breeding programs and necessary to find more regulatory ways to improve PS the mechanism through gene identification. Developing the PS resistant varieties are more acquiescent to mechanical harvesting and would provide an economical and long-term solution for rapeseed growers.

Genetic variation for dehiscence resistance exists naturally in *Brassica* germplasm (*B. rapa, B. napus, Brassica carinata* and *Brassica juncea*) [5, 6]; this can be helpful in plant breeding programs to improve the resistance against PS in commercial cultivars. The genetic network responsible for PS has been previously described in several studies [7, 8]. In brief, several transcription factors (TF) SHATTERPROOF1 and 2 (SHP1 and SHP2), INDEHISCENT (IND) and ALACTRAZ (ALC) are the main elements of the genetic network regulating the dehiscence zone (DZ) formation [8]. Two additional TFs, FRUUITFULL (FUL) and REPLUMLESS (RPL), are responsible for keeping the expression of DZ formation genes in particular area [9]. The cell wall thickening TFs (NST1 and 2) was previously described to regulate the genes involved in the cellulose and lignin synthesis, as *nst1* mutants were futile to form (lignified layer) LL in the DZ, not affecting the separation layer (SL) formation [10–12]. Recently, it has been reported that a TF, APETALA2 (AP2) negatively regulates the DZ formation genes (*SHP* and *IND*) and *REPLUMLESS* (*RPL*) to confirm the pertinent expression of all the genes in this network [13, 14]. Moreover, *FUL* together with *AUXIN RESPONSE FACTOR* (*ARF6* and *8*) limits the *AP2* activity in valves at posttranscriptional level revealing the role of microRNA (miR172) in Arabidopsis fruit development [8, 15]. Restricted cell growth was observed in the valves due to *AP2* activity, showing the similar phenotype with *ful* mutants [16, 17]. For normal dehiscence, both SL and LL have equal importance in the Brassicaceae pods, as LL exerts mechanical force to break the SL.

Several enzymes are also important to dissolve the SL and facilitate the whole mechanism together with other factors. The members of the PG family of pectin-degrading enzymes present downstream of the *ALC* and facilitate the formation of SL at maturity [8, 18, 19]. Compared with wild-type, Arabidopsis (*ARABIDOPSIS DEHISCENCE ZONE POLYGALACTURONASE*) *adpg1* mutants showed pod shatter resistance unless external mechanical pressure was applied to the siliques [18, 20, 21]. By contrast, *adpg2* single mutant appears to shatter normally while *adpg1 adpg2* double mutant remain intact even after applying mechanical force [20]. Despite its importance, the expression of *ADPG2* itself is not enough for cell separation [20]. Together with ADPGs pectin methylesterases also accompany the formation of DZ and possibly contribute to the degradation of middle lamella at valve separation [22, 23]. The absence of *ADPG1/2* and *NST1* expression in *ind* mutants, referring that they are direct or indirect targets of *IND* [10, 18]. However, the cell wall modeling pathways and upregulation of lignification pathways are very ambiguous, could be spatially separated into adjacent domains if both were regulated by *IND* still unclear, but probably mediated by some other unknown factors.

Therefore, unrevealing the PS regulatory mechanism in rapeseed would explore the foundation for attaining high-quality germplasm for breeding purposes. Thus, a pair of extreme accessions with a significant difference in silique shatter resistance index (SSRI) were used to compare the PG activity, lignin deposition and transcriptome analysis to discover the hidden molecular mechanism of PS in *B. napus*. By *K*-means clustering and co-expression analysis, we found that senescence-associated genes (*SAG*), and *PG* genes significantly accompanying with this trait and may provide insight into the regulatory mechanism of PS in *B. napus.* This study offers further information to understand the genetic basis of PS mechanism in *B. napus* well.

## Results

### Extreme materials are selected by measuring PS-related parameters

**Fig. 1:**
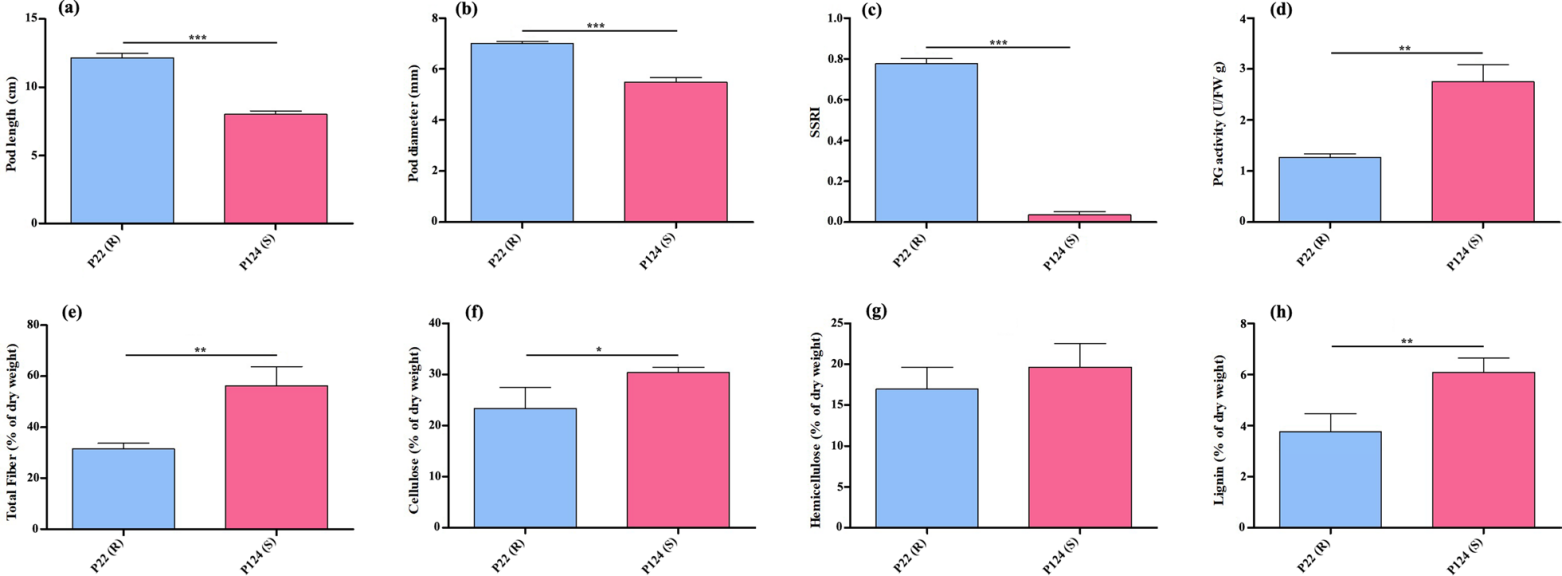
Phenotypic and cell wall component comparison between two *B. napus* accessions. **a** Comparison of pod length (cm) after harvesting between P22 and P124. b Comparison of SSRI. **c** Comparison PG enzyme activity at 49 DAP.** d–g** Comparison of cell wall components as total fiber, cellulose, hemicellulose and lignin, respectively. Representing values are the mean values with ± standard error (SE) of three replications. Asterisks on the error bars are presenting *P*-values (*P < 0.05; **P < 0.01; ***P < 0.001)

Both accessions (P22 and P124) were significantly different in pod length, diameter, PG activity and cell wall components (total fiber, cellulose, hemicelluloses, and lignin) (Fig. 1, Additional file 1: Fig. S1). The pod length (cm) and diameter (mm) in P22 were significantly higher (mean value and standard deviation, 12.16 ± 0.32 and 7.01 ± 0.10) than that of P124 (7.23 ± 0.25 and 5.48 ± 0.18), respectively (Fig. 1a–b). The trait-specific parameters SSRI and PG enzyme activity indicating the susceptibility of P124 such as SSRI (P22: 0.78 ± 0.075 and P124: 0.04 ± 0.015) and PG activity (P22: 2.75 ± 0.06 and P124: 1.75 ± 0.34) were highly significant in both accessions (Fig. 1c–d). Likewise, cell wall components were also significantly different in both accessions except hemicellulose as total fiber (P22 = 31.66 ± 2.08; P124 = 56.33 ± 7.50), cellulose (P22 = 23.33 ± 3.05; P124 = 30.33 ± 1.15), hemicellulose (P22 = 17 ± 2.65; P124 = 19.67 ± 2.89) and lignin (P22 = 3.96 ± 0.07; P124 = 6.1 ± 0.55) (Fig. 1e–h). These results state that cell wall components, especially lignin play a pivotal role coupled with PG enzyme to control the PS at terminal development stages.

### RNA-sequencing analysis

We collected 36 silique samples from two accessions at six developmental stages to predict the transcriptional network and identify important regulatory genes involved in the PS mechanism. Transcriptome sequencing produced a total of 219.57 Gb raw data, containing 1463.69 million reads, with an average of 6.09 Gb and 44.66 million reads respectively, per sample (Additional file 9: Table S1). Filtering the raw reads removed 1.47 Gb contaminant and low-quality reads, with an average of 40.62 million reads in each sample. The Q30 was varied from 94.49% to 92.80%, and the G + C percentage ranged from 46.72% to 49.41%. Unique mapping rates of all the samples were > 80% with an average of 87.64%, implying the reliability of our sequencing data to identify the DEGs and construction of the regulatory network.

The authenticity of all the samples was confirmed by correlation analysis as they presented high reproducibility (*r*^*2*^ > 0.95) among the biological replicates of each sample (Additional file 2: Fig. S2). The correlation among samples collected at different pod development stages was much lower than those among biological replicates. Moreover, the correlation pattern was quite different in the last four stages of P124 with the rest of the samples in both accessions, indicating the crucial role of those development stages (28–49 days after pollination, DAP) in the susceptibility of P124 (Additional file 2: Fig. S2b–d).

### Identification and classification of DEGs

**Fig. 2:**
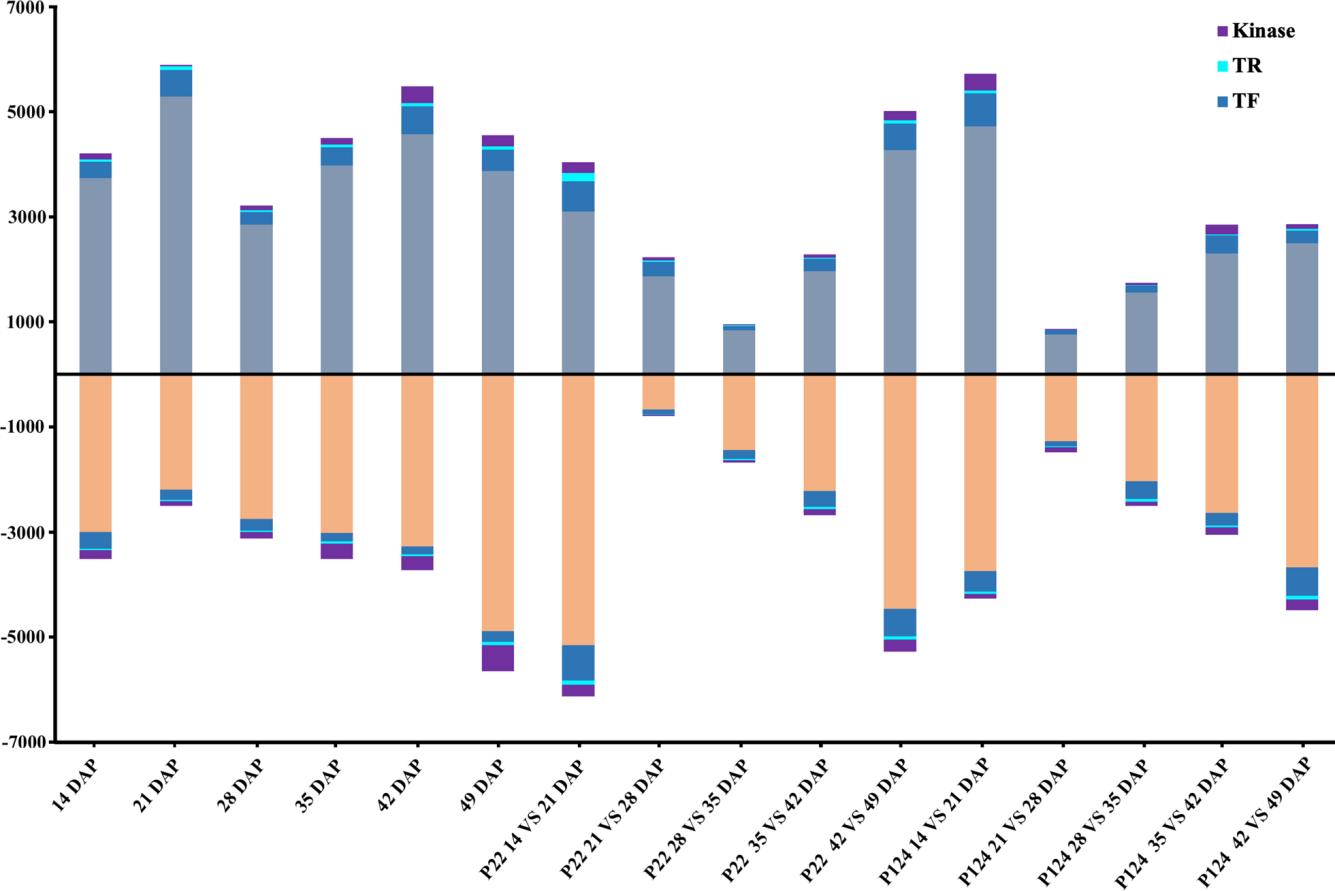
Classification of DEGs into TFs, TRs and kinases

Based on a comparison among six pod developmental stages between accessions and the adjacent stages, we identified a total of 10,973 unique DEGs (Fig. 2, Additional file 10: Table S2). In this comparison, the number of DEGs was highest (total = 10,201; up = 4553; down = 5648) at 49 DAP between pod shatter prone and resistant accessions, conferring the variation in pod transcriptome during the late development stages as pods attain maturity and move toward harvest (Fig. 2). This information is consistent with our PCA results suggesting that the whole transcriptomic reprogramming significantly occurred at late pod development stages especially at 49 DAP and might be the most crucial time to prepare the pods for upcoming harvesting and shattering events.

In comparing adjacent development stages of P22, the highest number of DEGs (10,286) were observed at 42–49 DAP and the number of upregulated and downregulated DEGs were almost the same. A similar trend was observed in P124 as the second highest DEGs were found between 42 and 49 DAP. While in comparison of 42–49 DAP between both accessions, the DEGs were more in P22 than P124. The number of upregulated and downregulated DEGs in P22 were almost the same, but the number of upregulated DEGs (5,725) in P124 were higher than that of downregulated genes (4,490) (Fig. 2; Additional file 10: Table S2). In relevance to our transcriptome data among pod development stages, the number of DEGs showed different pattern during 42–49 DAP, suggesting clear variation between both accessions in terms of upregulation, downregulation, and the total number of DEGs between them.

We classified DEGs into TRs, TFs and kinases using the online tool iTAK to understand which group is more important in PS. According to the results, *B. napus* consists of a total 6,146 TFs, 1242 TRs and 3424 kinases (Additional file 11: Table S3). Among DEGs, we found 3084 unique TFs, representing the transcription of TFs (*p* < 0.001) was regulated to a higher degree throughout the pod development than other regulatory genes, acquired from the Chi-square test between differentially expressed TFs and DEGs versus all TFs and all *B. napus* genes. Among differentially expressed TFs, the AP2/ERF-ERF, MYB, NAC and bHLH families appear to play a prominent role during pod development as 331, 269, 239 and 219 members of these families were correspondingly observed (Additional file 3: Fig. S3). Likewise, the expression of 522 unique TRs and 1559 unique kinases was equally significant (*p* < 0.001) as TFs and might have an important role in pod development. These results showing that TF, TR, and kinase are more important than other structural genes in regulating the PS*.* Remarkably, kinases (*p* < 0.001) were significantly higher at 49 DAP than TFs and TRs, indicating that post-translational regulation might be more important for PS in *B. napus.*

### GO enrichment analysis of DEGs

GO enrichment analysis of DEGs found that senescence, auxin transport and lignin accumulation might be responsible for PS in rapeseed (Additional file 12: Table S4). Since the upregulated genes at 42 and 49 DAP revealed the significant enrichment of “lignin metabolic process” (GO:0009808). Considering that PS varied significantly between both accessions, we assumed that transcriptional change of these genes might have an important role in this trait. Another group of upregulated genes may involve in PS by interacting with cell fate and water content, as they exhibited enrichment in “negative regulation of programmed cell death” (GO:0043069) and “response to water deprivation” (GO:0009414). Among downregulated genes important trait-specific genes could be observed at different development stages. GO terms at 42 DAP were “lignin biosynthetic process” (GO:0009809), and “auxin transport” (GO:0060918). At 49 DAP important GO terms among DEGs were “senescence” (GO:0010149), and “ethylene-mediated signaling pathway” (GO:0009873).

Similarly, GO enrichment analysis of DEGs at adjacent stages in each accession showed that the upregulation of senescence-related genes was quite similar in both. However, it started from an earlier stage in PS prone accession (P124). The upregulated GO terms revealed the “senescence” (GO:0010149), “regulation of programmed cell death” (GO:0043067) “lignin metabolic process” (GO:0009808), “abscisic acid-mediated signaling pathway” (GO:0009738) and “ethylene-mediated signaling pathway” (GO:0009873)-related genes in P22 and P124 accessions but the upregulation of these genes was noted at higher extent in P124 from earlier stage (28–35 DAP). However, negatively regulated GO terms were “water transport” (GO:0006833) and “cytokinin-mediated signaling pathway” (GO:0009736) observed in last two adjacent stages of both accessions (Additional file 13: Table S5). These results revealed that both *B. napus* accessions followed the same GO terms but differed in expression duration to control the PS and imminent events.

### Expression pattern analysis of DEGs by K-means clustering

**Fig. 3:**
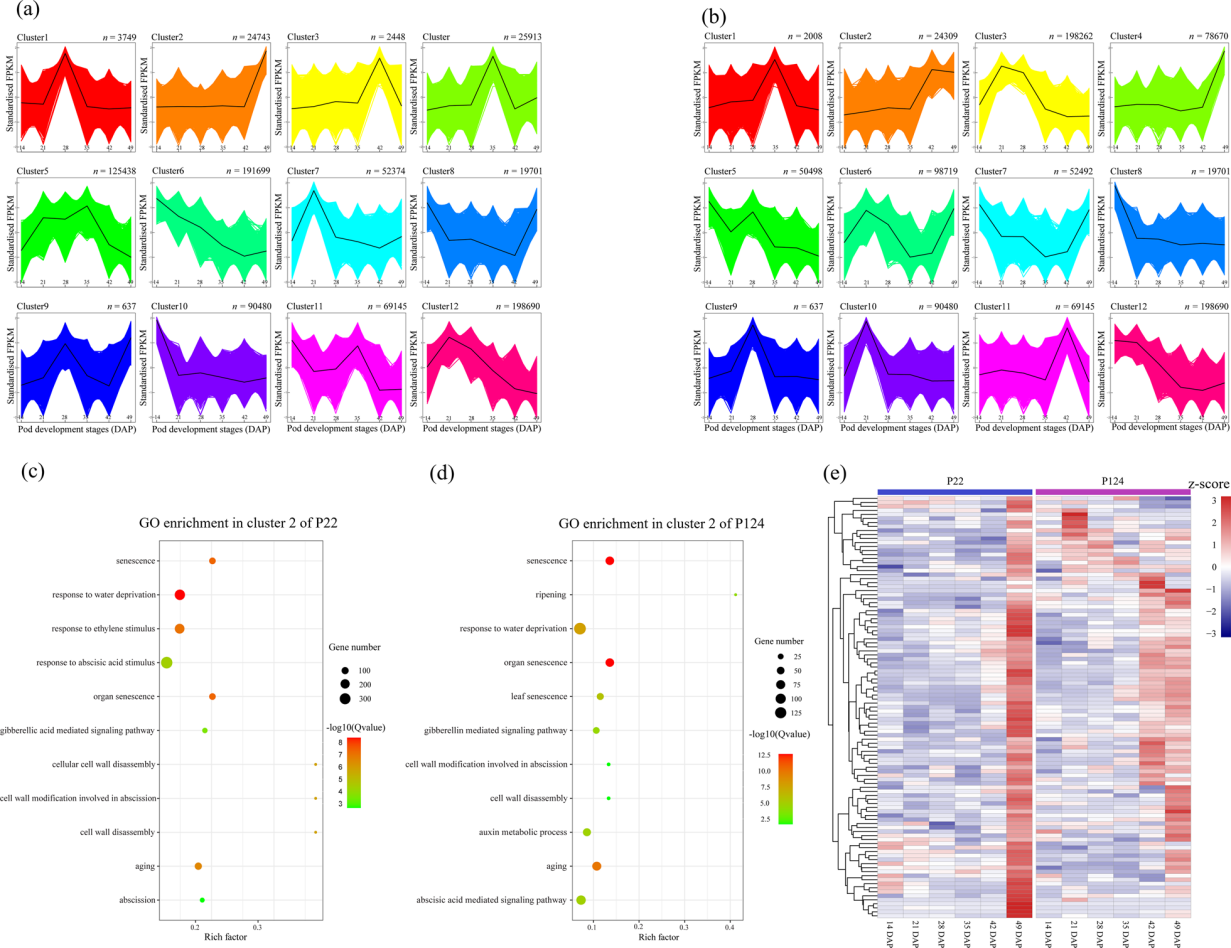
*K*-means clustering of DEGs. **a**, **b** Twelve clusters of P22 and P124, respectively, based on Log_2_(FPKM + 1) values at six pod developmental stages, on x axis developmental stages and on y axis FPKM values. **c**, **d** GO enrichment analysis in P22, and P124, respectively. **e** Heatmap of expression of senescence-related genes

To further investigate the PS regulation at different development stages, DEGs were subjected to *k-means* clustering and found that lateral pod development stages are crucial in governing the PS mechanism. Based on the expression pattern among all DEGs, 12 clusters were obtained in each accession (Fig. 3a–b). We observed a similar gene expression pattern in few clusters followed by the pod dehiscence pattern, such as cluster 2 in both accessions (P22 and P124) showed similar trend of gene expression toward the harvesting stage. GO and KEGG enrichment analysis showed that these genes were enriched in senescence, cell wall disassembly, water deprivation and hormonal activity. These results are consistent with GO analysis of DEGs among adjacent stages and between accessions (Additional file 13: Table S5), suggesting the role of senescence, hormone, and lignin deposition in PS. In P22, the expression of senescence-related genes was higher at 49 DAP but in the P124 expression level of those genes was started to increase from 42 DAP (Fig. 3e), representing the susceptibility of P124 against PS*.*

Comparative analysis by Heatmap displayed a similar pattern with *k*-means clustering and showed high transcription at 49 DAP in P22, but in P124 high expression was recorded from 35 DAP to upcoming stages (Fig. 3e). These results indicate that the degree of expression and time may contribute to the difference of *B. napus* resistance against PS trait and need to be explored further.

### WGCNA analysis and co-expression network construction

In crop improvement, TFs are foremost candidates to genetically improve the complex traits as they are the major regulator of a group of genes. To explore the co-expression networks accompanying the PS, we used R WGCNA software with an expression matrix in fragments per kilobase per million mapped (FPKM) and two different phenotypic accessions P22 and P124. The coefficient of correlation between samples and accessions represented high reliability among the biological replications hence all the outliers are present in the analysis (Additional file 4: Fig. S4). According to the results with those from the correlation coefficient, representing the pod samples from 35 to 49 DAP were grouped in the same clade (Additional file 4: Fig. S4a), suggesting the role of these developing stages in PS resistant and susceptible accessions.

**Fig. 4:**
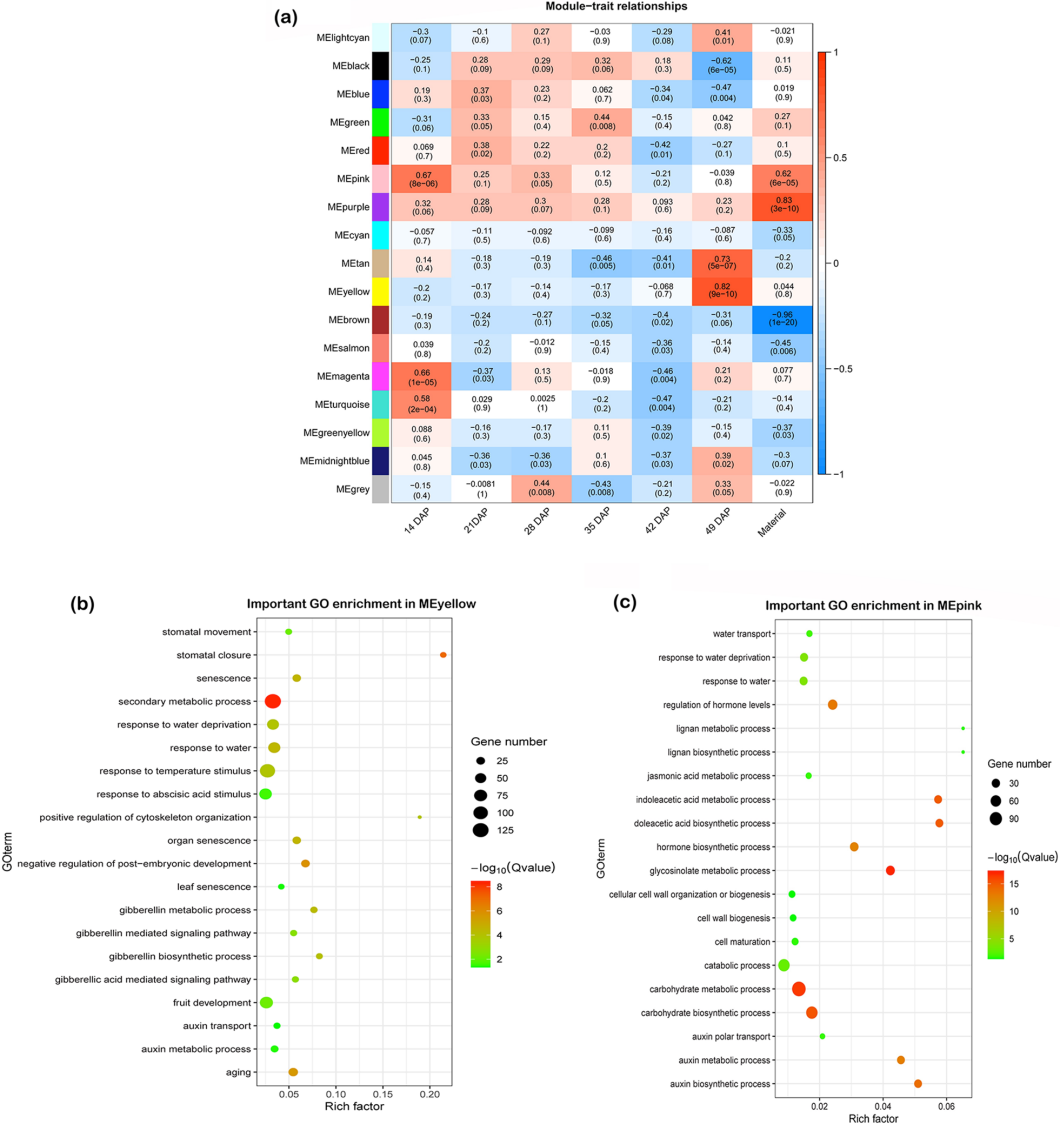
Module trait relationships and GO enrichment of selected color modules. **a** Heatmap representing the correlation between 17 color modules and two materials, with correlation coefficient and P value; **b**, **c** GO enrichment analysis of genes in MEyellow and MEpink modules, respectively

In WGCNA analysis (pickSoftThreshold) the optimal threshold was 18 at 0.9 fitting curve (Additional file 4: Fig. S4b). Then, we identified co-expression modules using the automatic blockwiseModules to construct the expression network (Additional file 4: Fig. S4c). This network construction of helps the color module visualization, indicating the highly and weakly correlated genes with different color schemes (Additional file 4: Fig. S4d). Our color module construction analysis showed that functional modules were clearly divisible. This analysis produced 17 color modules after combining the modules with the same expression pattern, each module contained similar expression pattern with respect to the stages and materials (Fig. 4).

According to the color module results pod developmental stages (14–49 DAP) and extreme materials (P22 and P124) demonstrated a significant positive and negative correlation with color modules. Most importantly color modules between materials and at 49 DAP were highly significant suggesting the trait variation and difference in gene expression at the end of the pod development stage. Then we analyzed GO enrichment for each color module (Additional file 14: Table S6), and selected modules with the highest correlation and relevance with the target trait. The highest correlation was found in MEbrown (*r*^2^ = − 0.96, *p* = 1E−20) but lacked any important GO term. However, genes in MEpink (*r*^2^ = 0.62, *p* = 6E−5) were significantly enriched in “lignin biosynthetic process” (GO:0009809) and “response to water deprivation” (GO:0009414) (Fig. 4; Additional file 14: Table S6) showed that the important genes responsible to PS present in this module. The MEyellow module from 49 DAP showed the highest correlation (*r*^2^ = 0.82, *p* = 9E−10) with PS-related GO terms such as “senescence” (GO:0010150), “stomatal closure” (GO:0090332) and “response to abscisic acid stimulus” (GO:0009737). These results are consistent with our cell wall component measurements as higher lignin might be deposited due to the higher expression of lignin biosynthesis genes and may lead to the higher susceptibility. Moreover, the regulation of senescence-associated genes and water availability has a crucial role in pod development and may interact with each other to cause PS in *B. napus*.

### Co-expression networks and hub genes regulating PS

**Fig. 5:**
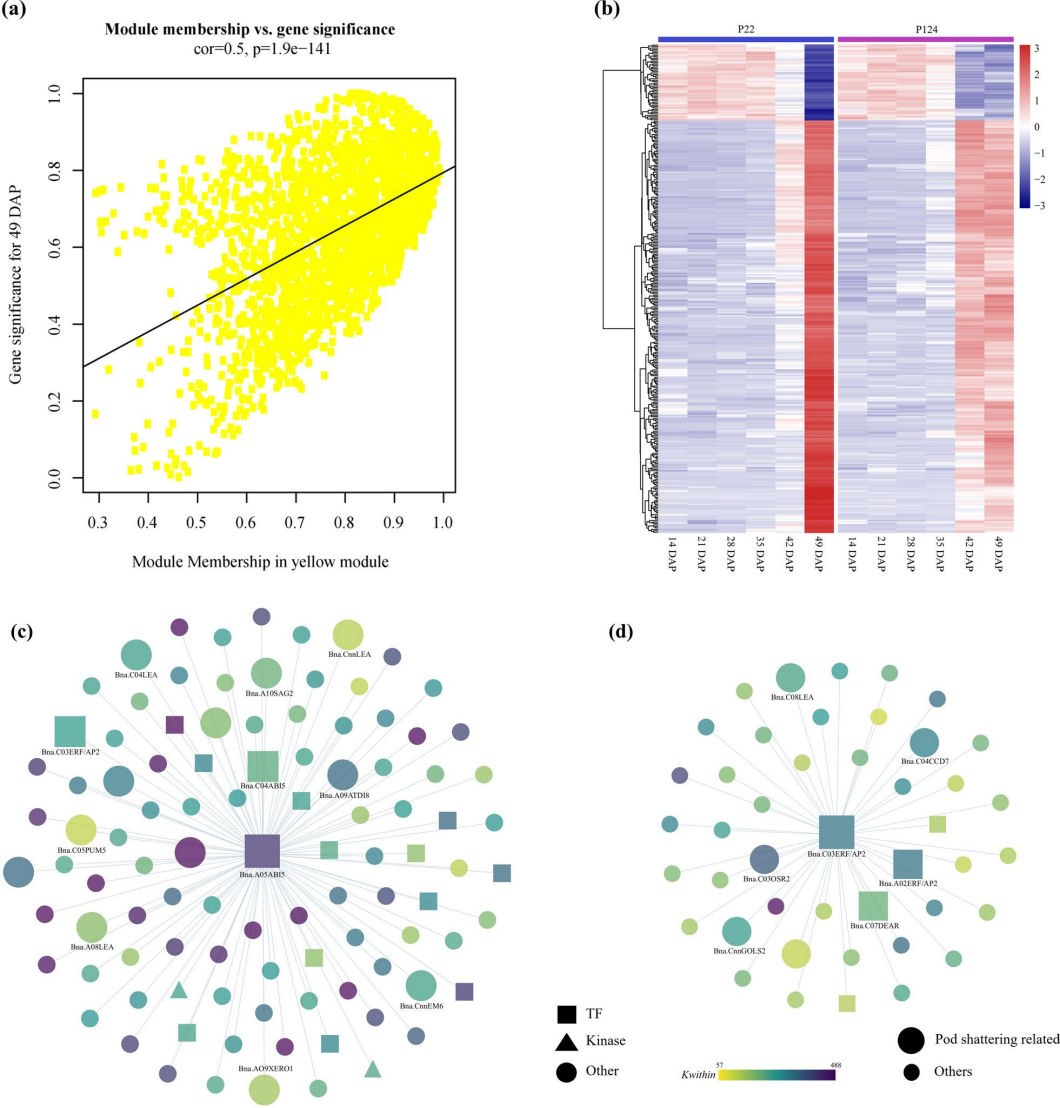
The WGCNA MEyellow module significantly correlated with PS mechanism. **a** Scatter plot representing correlation of module membership, correlation coefficient drawn between genes with 49 DAP. **b** Log_2_(FPKM + 1) normalized heatmap of MEyellow module genes (senescence related) of *B. napu*s. **c**, **d** Co-expression network of *Bna.A05ABI5* and *Bna.C03ERF/AP2-3*, respectively. TFs, TRs and Kinases are represented by different shapes

Analysis of the co-expression network showed that PS was highly correlated with MEyellow module (*r*^2^ = 0.82, *p* = 9E−10) (Fig. 4). The expression profile of most of the genes was greatly correlated with PS and the module eigengene in the MEyellow module (Fig. 5). MEyellow possessed genes participated in “senescence” (GO:0010150), “stomatal closure” (GO:0090332) and “response to abscisic acid stimulus” (GO:0009737) (Additional file 15: Table S7). Then, we constructed Heatmap based on the expression values of MEyellow module genes, and it showed that the module eigengene of that module was analogous with the average expression level of cluster 2 of each P22 and P124 from the *K-*means clustering results (Fig. 3a–b and e), implying that the PS trait in *B. napus* was mostly regulated by senescence-related genes.

We identified hub genes in important modules by assessing gene connectivity (*K-*within) based on absolute Pearson’s correlation; genes with the top 20% (*K*-within) value were selected as hub genes in the MEyellow module. In the selected 334 genes (MEyellow) *K*-within were ranged from 521.72 to 354.14, and those genes were classified into 39 TFs and 5 kinases based on iTAK results (Additional file 11: Table S3). Then we selected 2 TFs as hub genes from the MEyellow module, having highest *K*-within values, *Bna.A05ABI5* (*Bna.A05g08020D*, *K-*within = 379.15) and *Bna.C03ERF/AP2-3* (*Bna.C03g09040D*, *K-*within = 422.50). Their Arabidopsis ortholog genes are *At.ABI5* (*AT2G36270*, TF of bZIP) and *At.ERF/AP2* (*AT5G18450*, TF of DREB family), respectively. In *Bna.A05ABI5* network, *ABI5* was co-expressed with 101 genes comprising 14 TFs and 2 kinases (Fig. 5c). This network includes nine LEA proteins, ethylene insensitive response factor (*Bna.A06EIN*), senescence-associated genes (*Bna.A10SAG2*), water deprivation gene (*Bna.A09XERO1* and *Bna.C08 XERO1*), kinase control stomatal regulation (*Bna.A05CBC1*) and other genes which might regulate the lignin deposition in LL and its water contents. In the primary network of *Bna.C03ERFAP2-3*, 34 genes were co-expressed with it, including 3 TFs (2 DREB and 1 PLATZ family) (Fig. 5d). This network includes cell separation-related gene (*Bna.C03OSR2*), AP2 domain containing genes (*Bna.C07DEAR* and *Bna.A02ERF/AP2-4*), and two LEA proteins (*Bna.C08LEA4-1* and *Bna.CnnLEA*), those genes might have role in both SL and LL formation. Most of the genes in these networks had higher expression level during the late pod development stages (42–49 DAP), but P124 showed prolonged expression than P22 to cause susceptibility in this accession. Furthermore, this trait is controlled by the genetic variation and partially affected by the pod development and terminal stages.

### Comparative analysis of PS regulatory genes

The expression of PS regulatory genes has vital role in this mechanism. Our analysis suggested some key differences in the expression profile of these genes in *B. napus*, which might be the reason of a significant change in PS. Here we compared these genes by subjecting the expression values to log_2_ (FPKM + 1) and then normalized by *Z*-score.

**Fig. 6:**
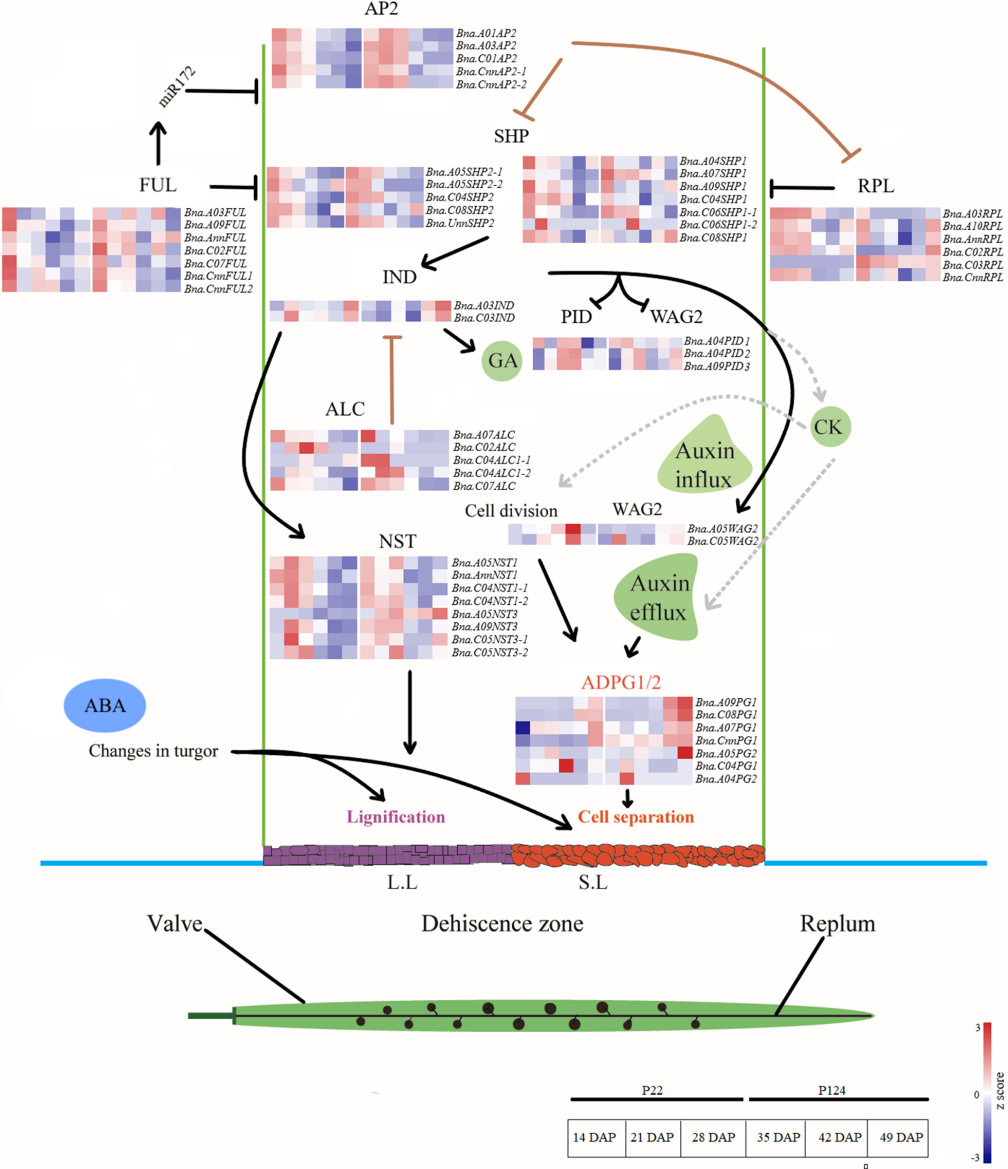
The heatmap of PS key genes in two *B. napus* accessions. PS mechanism, expression of both accessions was visualized by Log_2_(FPKM + 1), pod was divided into three parts valves, replum and DZ, denoted by different colors

According to the previously described PS mechanism *B. napus* contained 61 homologous genes (Fig. 6; Additional file 16: Table S8). Among these, four genes (two *BnaPG2*, *Bna.C09SHP1* and *Bna.A04ALC*) were not expressed at any stage. Most of the *FUL* genes have similar expression pattern apart from *Bna.AnnFUL* that highly expressed in P124 at the last sampling stage. In comparison, most of the *RPL* genes were highly expressed at 49 DAP in P124, which might be important in the differentiation of DZ. The antagonistic expression of *AP2* and *RPL* in the early development of pods is possibly involved in the regulating downstream *SHP* genes to facilitate the differentiation process. In contrast, higher expression of *IND* genes was observed during the late development stages, reflecting its role in the differentiation of DZ into LL and SL. In downstream of *IND*, *Bna.PID2-3* presented higher expression in P124. However WAG2 genes indicated high transcript levels in P22 at the last two stages which possibly caused the lower expression of *ADPG1/2* by its auxin transport activity. These results indicate the possible role of auxin in regulating pectin degrading enzymes. Among PG subfamily of polygalacutronases, four *PG1* genes (*BnaA09PG1, BnaA07PG1, BnaC08PG*1 and *BnaCnnPG1*) and one PG2 gene (*BnaA05PG2*) were highly expressed in susceptible accession at 49 DAP. Though in resistant accession all *PG1* genes exhibited the same pattern but lower expression level apart from *PG2* which showed a very low transcript level at the last sampling stage, indicating its important role in DZ formation. Therefore, our data is indicating that PS regulatory genes are conserved in their function in the Brassicaceae family.

### Role of lignin biosynthesis in PS

In relevance with our cell wall component analysis the difference in PS between *B. napus* accessions might be triggered by lignin deposition. In this pathway, 175 homologous genes were involved in *B. napus,* of which eight genes were not expressed in any sampling stage (Additional file 6: Fig. S6; Additional file 17: Table S9). The *PAL* and *C4H* genes were highly expressed in resistant material, while *4CL* had higher expression level in P124 at 49 DAP. The expression level of *HCT* and *C3’H* genes was higher at 35–49 DAP in P22 and that genes present in the cluster 8 of P22 accession. The expression pattern indicated that lignin biosynthesis genes remained active even near to harvest stage in P22 that might be due to the longer pod size. While *Bna.CCoAOMT* genes were highly expressed in P124 during most of the developmental stages and may cause greater lignin deposition at the end of pod development. Lignin biosynthesis is a very complex and long process plays an important role in plants throughout their life cycle. Our results suggesting no clear variation in lignin biosynthesis genes except *Bna.CCoAOMT* coding genes. The expression of most of the genes was higher in P22 possibly due to the higher pod length but at the same time the expression of all the genes corresponding to different enzymes similar in P124 that reflect its higher accumulation of lignin which might results higher level of susceptibility (Fig. 1).

### Validation of sequencing data

For the validation of our RNA-seq data, we selected 12 genes for qRT-PCR analysis at six pod development stages. The relative expression values of DEGs showed a positive correlation with fold changes variation taken from transcriptome data (Additional file 7: Fig. S7). The correlation coefficient of RNA-seq and qRT-PCR for the 12 DEGs ranged from 0.90 to 0.78. Due to the silencing of some genes at different stages in both *B. napus* accessions, all the FPKM values were changed from zero to 0.001 for fold change validation. Only two genes showed less than 0.80 correlation coefficient, however, the rest of the genes presented better correlation, indicating the accuracy and reliability of our RNA-seq data.

### SSRI analysis of candidate genes

**Fig. 7:**
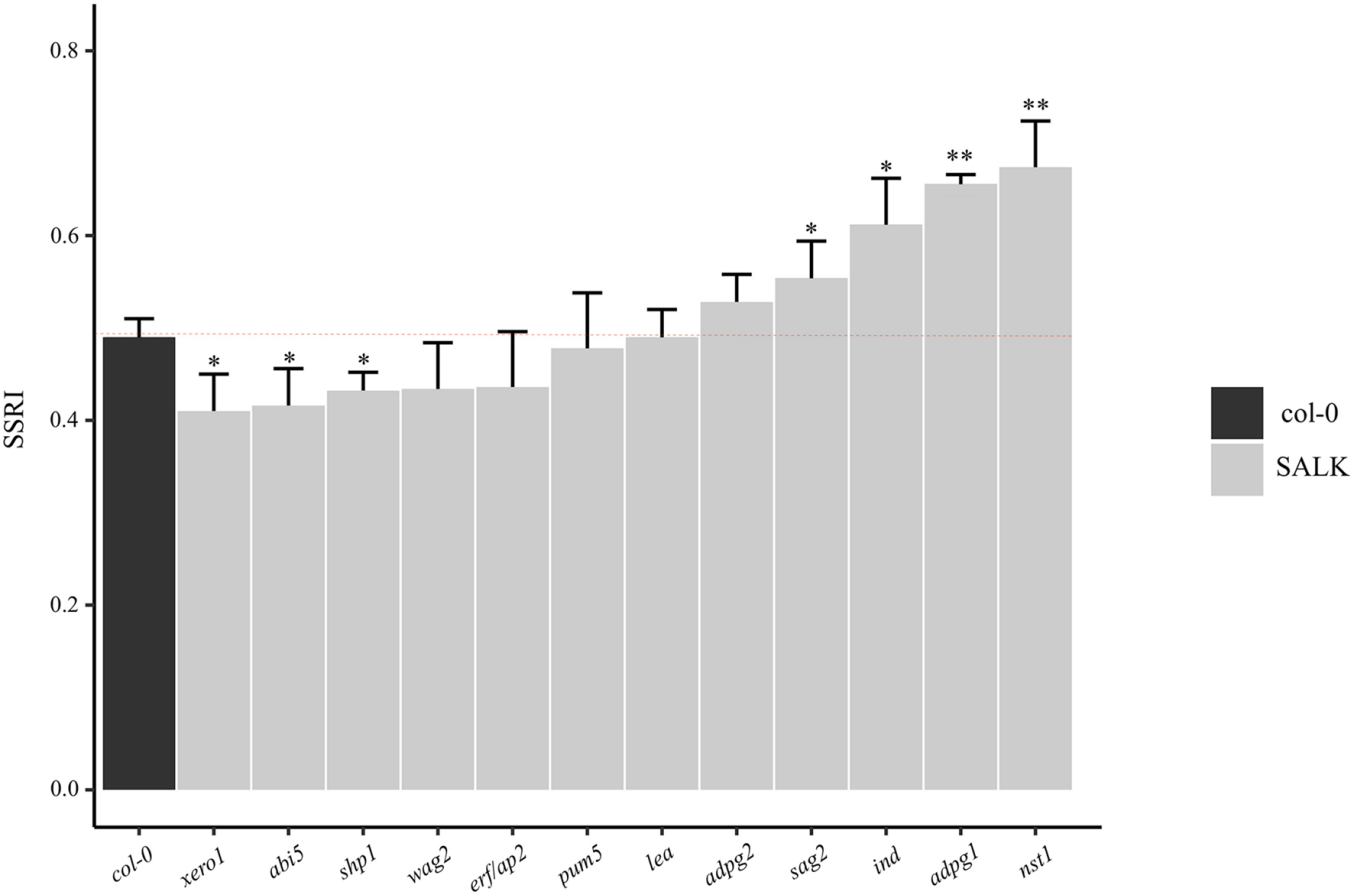
SSRI measurements of hub and marker genes in Arabidopsis. Red dotted line representing the 50% SSRI in WT used to compare with T-DNA mutants of twelve genes. Asterisks above error bars representing significant differences of T-DNA mutants Col-0 (Student’s *t*-test; *P < 0.05 and **P < 0.01)

To further explore the PS mechanism of PS marker and hub genes, we performed SSRI analysis of T-DNA lines. We selected six genes potentially involved in PS from our transcriptome analysis to perform the SSRI analysis together with six previously described marker genes. Genotyping and reduced gene expression of all the T-DNA lines was confirmed before SSRI measurements. The T-DNA mutants of four genes *NST1, ADPG1*, *IND* and *SAG2* exhibited significantly high SSRI at 37.7%, 34.1%, 24.5% and 12.2%, respectively, over the WT. Whereas mutants of three genes (*XERO1*, *ABI5* and *SHP1*) had significantly lower SSRI compared with WT as 16.3%, 14.3% and 12.2%, respectively. However, no significant change was observed in the remaining five mutant lines (Fig. 7). These results showing that *ABI5* might have important role in LL to increase the mechanical force required to break the SL during the PS mechanism. However, *SAG2* is possibly involved in SL formation together with PG activity in lateral pod development stages. Taken together, the SSRI measurements of hub and marker genes revealed that hub genes identified in this research might have a crucial role in the PS mechanism and need to be explored at molecular level.

## Discussion

### Senescence-associated genes may regulate PS

*B. napus* yield has always been a principal factor as PS upon harvesting causes severe yield losses. So, understanding the molecular mechanism of PS in *B. napus* can provide vital information for other crops in this family. Here, we studied this mechanism in two *B. napus* accessions with significant variation in pod shatter resistance. Our WGCNA co-expression network analysis revealed that the MEyellow module exhibited the highest correlation with PS (Fig. 4). The corresponding genes in this module were significantly enriched in the senescence-related events. In *K*-means clustering analysis, we found two important clusters with different expression profile but similar to their senescence-related functions. A comparative analysis between WGCNA and *K*-means clustering presented important genes associated with senescence, water deprivation, auxin transport and lignin deposition. All these genes were significantly correlated with PS, revealing that transcriptional regulation of senescence-related genes might be the potential target to improve crop yield by reducing the seed losses at harvesting time in Brassica crops.

PS is associated with the onset of senescence during the silique development and cell separation processes [24]; as a result, senescence-related gene play a crucial role in controlling PS in *B. napus.* We observed most of the senescence-related genes highly expressed at 42–49 DAP in P124, However, in P22 those genes only expressed at 49 DAP, reflecting the higher resistance due to the short duration of the expression profile (Fig. 3e). According to our results, *SAG2* gene plays an important role in senescence-related functions and regulated by the upstream bZIP TF, *ABA INSENSITIVE 5* (*ABI5*) (Fig. 5c). Consistent with previous results, the response of abscisic acid (ABA) associated to the desiccation of fruit dehiscence and facilitate the appropriate timing of PS [22, 24].

Researchers have previously described the role of *SAG* genes in dehiscence and programmed cell death (PCD) [25, 26]. Zhang et al., [27] described that *AtSAG12,13* genes have an important function in senescence-related processes (organ senescence, leaf senescence etc.) and are regulated by the growth hormones such as ABA and jasmonic acid (JA) [27]. Rapeseed has several SAG genes (2 *Bna.SAG1, Bna.SAG2*, 2 *Bna.SAG12, 3 Bna.SAG12* and *Bna.SAG20*) which were gradually and highly expressed toward the pod ripening. While both *BnaSAG1* genes were upregulated in P124 and might have crucial role in the PS process. The upregulation of senescence-related genes in susceptible accession from 42 to 49 DAP while in resistant accession from 49 DAP suggested that PCD and senescence leading to separation processes has started earlier in susceptible accession and are probably the reason for susceptibility.

### Role of AP2/ERF and ABI5 TFs in PS

**Fig. 8:**
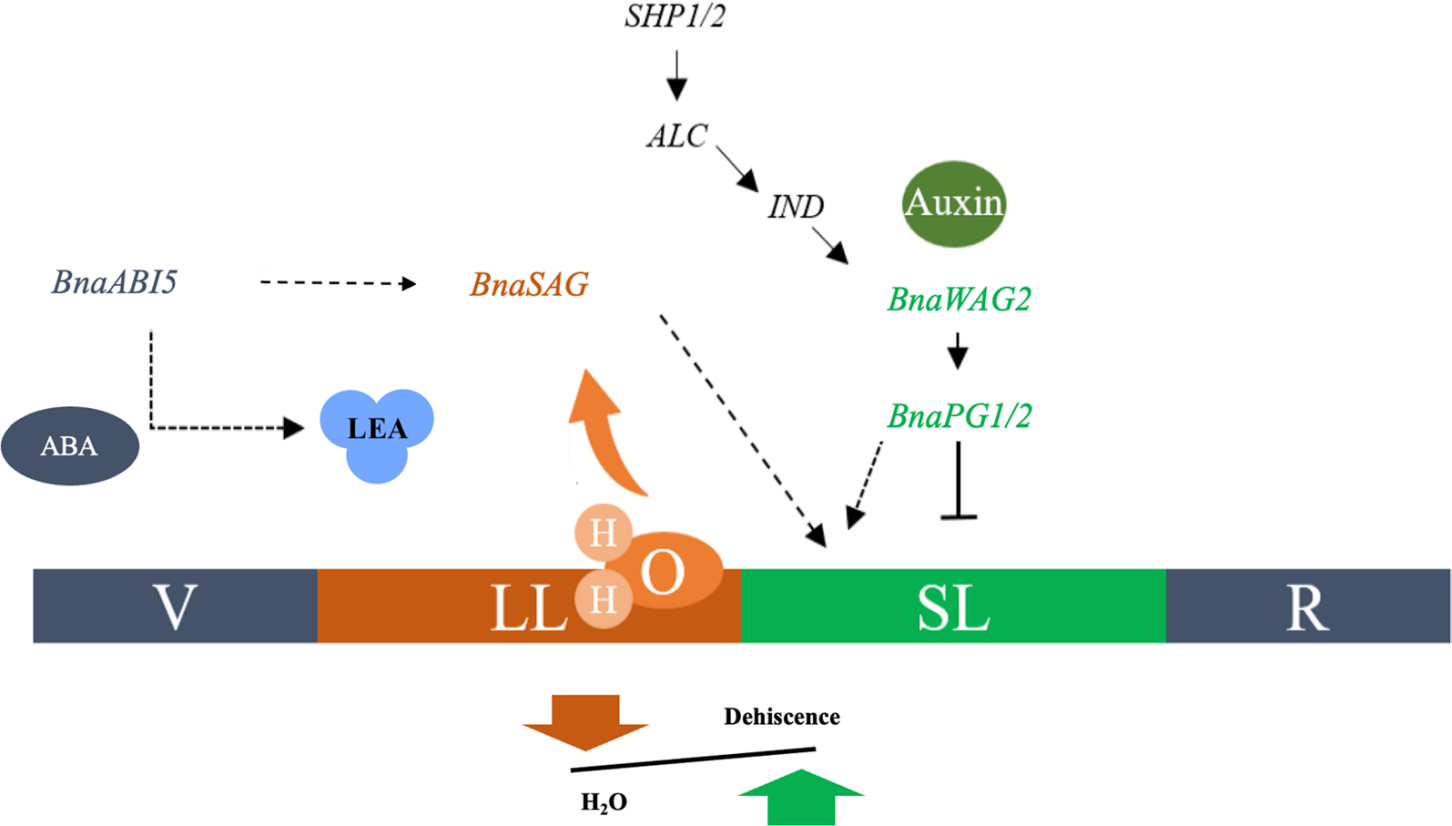
Proposed regulatory model in rapeseed pods. Proposed model by which *BnaABI5* regulate the senescence-associated genes, and auxin flux control the *BnaPG* activity to upcoming events in pod development. The decreasing moisture together with ABA regulation exacerbate the PS

In the PS mechanism, several TFs and growth hormones participate in regulating the LL and SL formation. Our WGCNA analysis identified the MEyellow module, which has the highest correlation with DZ formation and is significantly related to DZ formation genes (Fig. 5a). The two TFs *Bna.A05ABI5* and *Bna.C03ERF/AP2-3* were selected as hub genes in this network (Fig. 5c–d). *ABI5* was previously described to have a regulatory role in DZ formation and leaf senescence [28] capable of inducing the turgor change which ultimately results in dehiscence. In Brassica crops (*B. rapa* and *B. juncea*), variation in shattering resistance has been correlated with the fluctuations in pod dehydration at maturity [29] and remarkably governed by different ABA activity in the pods of these species [22]. We found several downstream targets of *Ban.A05ABI5* which are directly or indirectly involved in DZ formation by altering the water contents in LL. This network includes several LEA proteins, senescence-associated genes, and stomatal movement-related genes. Most of these genes cause a change in moisture in LL; as a result, it produces different flexibility to the lignin which can exert a mechanical force on SL ultimately promote the PS. In the DZ, *AP2* genes downregulates its downstream TF *SHP1/2*, which possibly decreases the activity of the PG enzyme in SL [8]. The evolutionary study of dehiscent and resistant fruits in Lepidium (*Brassicaceae*) revealed the possible function of *AP2* genes in downstream *IND* and *SHP1/2* [30, 31]. Here, we noticed that the AP2 domain (AP2/ERF) may regulate the transcription level of downstream TFs in the DZ, and the higher expression level of these genes is probably the reason for higher resistance in P22. Our results suggested that *ABI5* has a potential role in LL and controls its mechanical strength by water contents, while the AP2 domain containing genes (*Bna.ERF/AP2*) are possibly involved in the SL modulation by interacting with its downstream TFs (Fig. 8).

### Expression profile of DZ formation and lignin biosynthesis genes

The main mechanisms of the genetic network affecting the morphogenesis of the DZ in the *Brassicaceae* fruits have been well-studied for several years. In this study, we compare the expression profile of these regulatory genes in PS resistant and prone *B. napus* materials (Fig. 6). We observed that AP2 and RPL were highly and antagonistically expressed during early stages in both materials, and as a result, *SHP2* expression was high in susceptible accession which reflecting the higher downregulating effect of RPL on *SHP2* in resistant accession. Interestingly, we observed that both TFs in *Bna.C07 SHP1/2* were highly expressed in susceptible material at 49 DAP, suggesting an important role in resistance. The genetic variation of *B. napus* progenitors (AACC) against pod dehiscence was previously described, as *B. rapa* (AA) having higher resistance than the *B. oleracea* (CC) [5, 32, 33] which strengthen our results. We also found that the higher expression of *BnaPG1/2* genes in susceptible accession in mature pods and might be regulated by the upstream auxin transporter kinase (*BnaWAG2*)*.* The upregulation of *BnaPG1/2* genes accelerates the activity of cell wall loosening enzymes in *Brassica* crops [18, 34, 35]. However, in the lignin biosynthesis pathway there was no clear differentiation in expression patterns between both accessions except *CCoAOMT* genes, indicating that lignin is not a direct regulator of PS. However, variation in moisture can change the mechanical strength of LL by which it exerts more force on SL. However, the lignification and separation are two key components in this regulation and need to be explored further to verify the role of *ABI5* and *ERF/AP2* in this mechanism.

Henceforth, we proposed a model mechanism by which hormones and the external environment interact with each other to regulate the PS. The expression of *WAG2* regulates the PG activity and *ABI5* may communicate with their respective downstream genes together with changing water contents in the environment (Fig. 8). The decreasing moisture in pods increases the mechanical strength of lignin and facilitate the breakdown of SL through upregulation of *SAG* genes. Therefore, a deep understanding of lignin activity and cell wall dissociation events may foster the new insights to decrease the seed loss by PS in rapeseed.

## Conclusions

Here, we compare two accessions with significant PS variation at six pod developmental stages and identified 10,973 DEGs from transcriptome comparison. Both WGCNA and *K*-means clustering identified senescence-associated genes with positively correlated expression patterns in the PS mechanism. Further analysis reveals that TF *Bna.A05ABI5* accelerate the shattering phenomenon by regulating *Bna.A10SAG2* and *Bna.C03ERF/AP2* in DZ. We identified another TF *ERF/AP2* in the upstream of *Bna.A05ABI* that might be involved in the resistance against this trait by downregulating the *SHP1/2*. In previously described DZ formation mechanism, we observed higher expression of *Bna.C07SHP1/2, BnaPG1/2* in susceptible accession and *BnaWAG2* in resistant accession, which may have an important role in the PS mechanism. Moreover, in the lignin biosynthesis pathway we could not find obvious differentiation except for *CCoAOMT* genes. That shows that lignin regulation is not directly involved in PS; however, the fluctuation in moisture in LL probably changes the mechanical force required to break the SL during PS events. In addition, the PS mechanism is not only controlled at the genetic level but also partially regulated by the pod development and terminal stages. This study will reveal the comprehension of the genetic association architecture of PS in *B. napus* thus facilitating the breeders to improve germplasm by genetic engineering.

## Material and methods

### Plant materials

Seeds of two *B. napus* accessions, P22 (pod shatter resistant; R) and P124 (pod shatter susceptible; S) were obtained from the Chongqing Rapeseed Engineering Research Center (CRERC). Seeds were cultivated during the first week of October 2018–2019, one month later transplanted into the research field at Southwest University, China (29° 45′ N, 106° 22′ E, 238.57 m), and cultivated as previously described [36]. Depending on the phenotypic observations, approximately similar time duration (*R* = 55 and *S* = 58 days) from first flowering to the final maturation stage was observed between R and S.

### Phenotype measurements

Pods of both accessions at 49 DAP were harvested based on the previously described method, Biologische Bundesanstalt, Bundessortenamt, and CHemical (BBCH) industry scale [37]. For pod length and diameter measurements, six representative plants were chosen from the center of the plot. SSRI was measured in accordance with the previous method [38, 39], with slight modifications. First, all the samples (mature pods) were synchronized, at 22 ℃ ~ 25 ℃ and 50% humidity. Five replicates of pods were subjected to a closed polythene container height (14 cm), (diameter = 10 cm), with 50 steel balls (diameter = 8 mm), and shaken mechanically (IS-RDD3, Crystal Technology & Industries, Inc. America) with the frequency of 300 rpm/min. After each replication, the number of broken and unbroken pods was counted. The SSRI was calculated as the following equation:

$$SSRI = 1- \sum\limits_{i=1}^{5} \left(20-xi\right)/100.$$

The number of opened pods (damaged and broken) at each replication is denoted by *xi*.

### Cell wall component and PG activity

The cell wall components were measured with minor changes as previously described [40, 41]. For each accession (R and S), 10 g dried pods (mature) were grounded for cell wall components extraction. The analysis was replicated thrice with three biological replications of each. Protein for PG activity was extracted in agreement with the previous method [42], with few changes. Pods of both accessions at 49 DAP were subjected to the protein extraction for further analysis. PG activity was assayed followed by an earlier study [43]. PG activity was defined as the amount of enzyme required to produce 1 μg of polygalacturonic acid (GA) per hour per mg of proteins [18, 44].

### RNA sequencing and identification of DEGs

Pod samples were collected from 15 to 49 DAP with seven days of intervals. To compare the transcript changes, a total of 36 pod samples (2 accessions × 6 developing stages × 3 biological replications) were obtained. Total RNAs were extracted using an RNAprep Pure Plant Kit (Tiangen, Beijing, China), and sent to Novogene Corporation (Beijing, China) for transcriptome sequencing on an Illumina HiSeq 2500 platform. Low-quality reads, connectors, and barcode sequences were removed by Trimmomatic-0.39 [45]. Then STAR-2.5.3 was used to align the clean data to the *B. napus* reference genome v3.0 (http://brassicadb.org/brad/) [46]. Gene expressions were calculated as count number and FPKM followed by the previous method [47]. The correlation analysis among all sample was performed by principal component analysis (PCA), and the coefficient of correlation was examined through the R package ggfortify [48]. Finally, DEGs were found through the R package DEseq2, with a previously standardized method [49].

### Identification of TFs and genes related with PS mechanism

All the peptide sequences of *B. napus* were subjected to the online program iTAK v1.6, (http://itak.feilab.net) to categorize into transcription regulator (TRs), TFs and kinases [50]. Subsequently the shattering resistance differed significantly between R and S accessions, and we found all the genes involved in the lignin pathway of *Arabidopsis thaliana* [51–53], that might play a crucial role in shattering. Then a reciprocal BLASTP (*E*-value cutoff 1e-5) was used to search the homologous genes in *B. napus* against *A. thaliana* [54], and peptide sequences were scrutinized with pfam scan (https://www.ebi.ac.uk/Tools/pfa/pfamscan/) to further verify the respective functional domains.

### K-means clustering of DEGs

To identify the expression of genes for RNA-sequencing data, *K*-means clustering is an efficient tool [55]. To determine the expression pattern of DEGs with probable biological functions for our target trait (shattering resistance) cluster analysis was done by the *K*-means using the cluster package in R with Pearsons’ correlation distance. The best possible number of clusters was selected by the gap statistic and analyzed by the clusGap function in R package factoextra [56]. Then *K*-means clustering was done with 12 optimal clusters for each *B. napus* accession. All the heatmaps were drawn by the normalized expression values Log_2_(FPKM + 1), and *Z*-score normalization and heatmaps were created by the R package (pheatmap).

### Weighted gene co‑expression network analysis (WGCNA)

We established a co-expression network through the WGCNA package in R to identify the co-expression modules and important regulatory genes related to our target trait in *B. napus *[57]. In brief, we kept only those genes with FPKM values higher than one in any pod sample and then log2 normalized before the next step. The soft threshold power was ascertained by pickSoftThreshhold relying on the scale-free topology model fit (*R*^*2*^) > 0.9, then the blockwiseModules network construction method was employed to get the highly correlated modules for the trait of interest. Following parameters were used to perform to complete the analysis: power, 18; miniModuleSize, 50; TOM-type, unsigned; maxBlockSize, 35,000; and mergeCutHeight, 0.25. The online platform for Plant Transcriptional Regulatory Map (PlantRegMap, http://plantregmap.gao-lab.org) was adopted to perform the regulatory connections between TFs and their co-expressed genes in the network [58]. Then, the predicted regulatory network was shown by Cytoscap v3.7.1 [59].

### Gene Ontology (GO) and KEGG enrichment analysis

All the *B. napus* genes were annotated with BLASTP alongside the Arabidopsis proteome dataset (TAIR10) with the following parameters: E-value cut-off of 1E − 5 [54]. GO enrichment analysis was completed through the BiNGO plug-in function in the Cytoscape version v3.7.1 [59]. Significantly overexpressed GO terms were obtained with FDR < 0.05 threshold. Then, an online tool OmicShare (https://www.omicshare.com/tools), was used to complete the KEGG pathway analysis. Bubble plots for the KEGG pathway and GO terms were constructed with the R package (ggplot2) [60].

### Qualitative real-time time PCR (qRT‑PCR) validation

To validate the accuracy of our RNA-seq data and DEGs we performed qRT‑PCR analysis, the cDNA was synthesized from 11 μg of total RNA (used for RNA-sequencing) using a PrimeScript RT Master Mix Kit (TaKaRa, Dalian, China). A total of 12 genes (7 genes from the PS mechanism and 5 genes were senescence related) were chosen for the qRT-PCR assays. All the primers (Additional file 18: Table S10) for selected genes were obtained from the qPrimerDB database (https://biodb.swu.edu.cn/qprimerdb) [61]. All reactions were subjected to the Minimum Information for Publication of Quantitative Real-Time PCR Experiments (MIQE) instructions. Two internal controls (*Bna.ACT7* and *Bna.UBC21*) were used and relative expression values were obtained by the 2^*−ΔΔCt*^ method [36].

### SSRI analysis of candidate genes

For the SSRI analysis of candidate genes, T-DNA mutants, and wild-type Arabidopsis plants (Col-0) were used in this study. Twelve mutant lines were purchased from the Arashare database (https://www.arashare.cn) and genotyped by PCR for homozygous confirmation. All the information about accession numbers and primers is listed in the Table (Additional file 19: Table S11). Plants were grown under short-day conditions (10 h light/22 ℃) in a controlled environment. SSRI was measured according to the previous method with minor changes [62]. Mature pods were harvested and synchronized at 22 °C ~ 25 °C (50% humidity). Twenty intact synchronized pods (3–4 cm length) were put in a glass petri dish (diameter, 60 mm) with 6 steel balls (Ø 1 cm, 7.08 g). Five petri dishes were fixed on a shaker and set the frequency and time to get a 50% SSRI value using wild type. Then compared with mutant lines. Pods were considered as “shatter” when at least one valve was separated.

## Supplementary Information

**Additional file 1: Fig. S1.** The siliques of accessions P22 and P124. Scale bar indicates 1 mm.

**Additional file 2: Fig. S2.** PCA and coefficient of association analysis of both accessions (a) The 2D PCA analysis of P22 and P124, Same colors representing the biological replicates (b-c) Heatmap of all samples and correlation coefficient of P22 and P124, upper right corner representing relevance in the form of circles, and the bottom left is showing the correlation coefficient.

**Additional file 3: Fig. S3.** Classification of differentially expressed TFs.

**Additional file 4: Fig. S4.** The construction of co-expression network. (a) Clustering tree of all 36 samples; (b) Left one showing the relationship between soft threshold and scale independence. Right, one showing the relationship between soft threshold and mean connectivity; (c) The cluster dendrogram; (d) The correlation among all color modules represented in the form of heatmap, darker colors representing higher correlation.

**Additional file 5: Fig. S5.** The co-expression network of MEyellow module. Different shapes show TFs, TRs and kinases.

**Additional file 6: Fig. S6.** The heatmap of lignin biosynthesis pathway. Lignin biosynthesis pathway, expression of enzymes encoded genes at each step was represented by Log2(FPKM + 1) normalized values.

**Additional file 7: Fig. S7.** qRT-PCR validation of DEGs. Twelve DEG genes were selected for the qRT-PCR validation. FPKM and qRT-PCR values were represented with blue and orange color, respectively. The regression (r2) coefficients were presented on the left top of each gene

**Additional file 8: Fig. S8.** The relative expression level WT and T-DNA mutants candidate genes. Data representing means and standard deviation (Student’s t-test; *P < 0.05, **P < 0.01).

**Additional file 9: Table S1.** Summary of transcriptomic data and mapping competence.

**Additional file 10: Table S2.** Classification of DEGs identified at different development stages and between adjacent stages.

**Additional file 11: Table S3.** Identification and classification of all TRs, TFs and kinase genes in B. napus.

**Additional file 12: Table S4.** GO enrichment analysis of DEGs found by comparison of two accessions at the six stages.

**Additional file 13: Table S5.** GO enrichment analysis of DEGs found by comparison of adjacent stages in each accession.

**Additional file 14: Table S6.** GO enrichment analysis of seventeen color modules in WGCNA.

**Additional file 15: Table S7.** GO enrichment analysis of P22 and P124 clusters.

**Additional file 16: Table S8.** Expression values of pod shattering regulatory genes at six developmental stages in two B. napus accessions.

**Additional file 17: Table S9.** Expression values of lignin biosynthesis genes at six developmental stages in two B. napus accessions.

**Additional file 18: Table S10.** qRT-PCR primers for representative DEGs.

**Additional file 19: Table S11.** Functional analysis of T-DNA mutant lines.

## Data Availability

The raw sequencing data were submitted in the BIG data center (BIGD) under Bio-Project accession number PRJCA008288.

## Abbreviations

PS: Pod shattering
PG: Polygalacturonase
WGCNA: Weighed gene correlation network analysis
TF: Transcription factor
SHP1/2: SHATERPROOF1/2
IND: INDEHISCENT
ALC: ALACTRAZ
DZ: Dehiscence zone
FUL: FRUUITFULL
RPL: REPLUMLESS
LL: Lignified layer
SP: Separation layer
ARF: AUXIN RESPONSE FACTOR
ADPG1/2: ARABIDOPSIS DEHISCENCE ZONE POLYGALACTURONASE1/2
SAG: Senescence-associated genes
CRECRC: Chongqing Rapeseed Engineering Research Center
GA: Polygalacturonic
PCA: Principal component analysis
DEG: Differentially expressed genes
GO: Gene Ontology
qRT‑PCR: Qualitative real-time time PCR
MIQE: Minimum Information for Publication of Quantitative Real-Time PCR Experiments
ABA: Abscisic acid
ABI5: ABA INSENSITIVE 5
PCD: Programmed cell death
JA: Jasmonic acid
*B. napus*: *Brassica napus*
*A. thaliana*: *Arabidopsis thaliana*
