# Supplementary figures and images for: Comparative transcriptome and co-expression network analysis revealed the genes associated with senescence and polygalacturonase activity involved in pod shattering of rapeseed
Source: Biotechnol Biofuels Bioprod. 2023 Feb 7;16:20. doi: 10.1186/s13068-023-02275-6 (PMC9906875; doi:10.1186/s13068-023-02275-6)

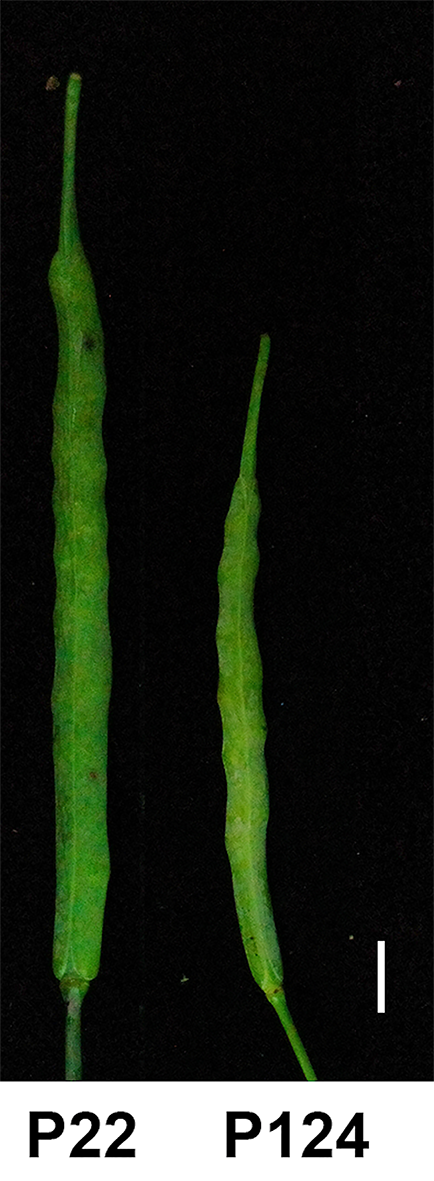

Supplement: Supplementary file 1 — Additional file 1: Fig. S1. The siliques of accessions P22 and P124. Scale bar indicates 1 mm. [file 13068_2023_2275_MOESM1_ESM.tif]

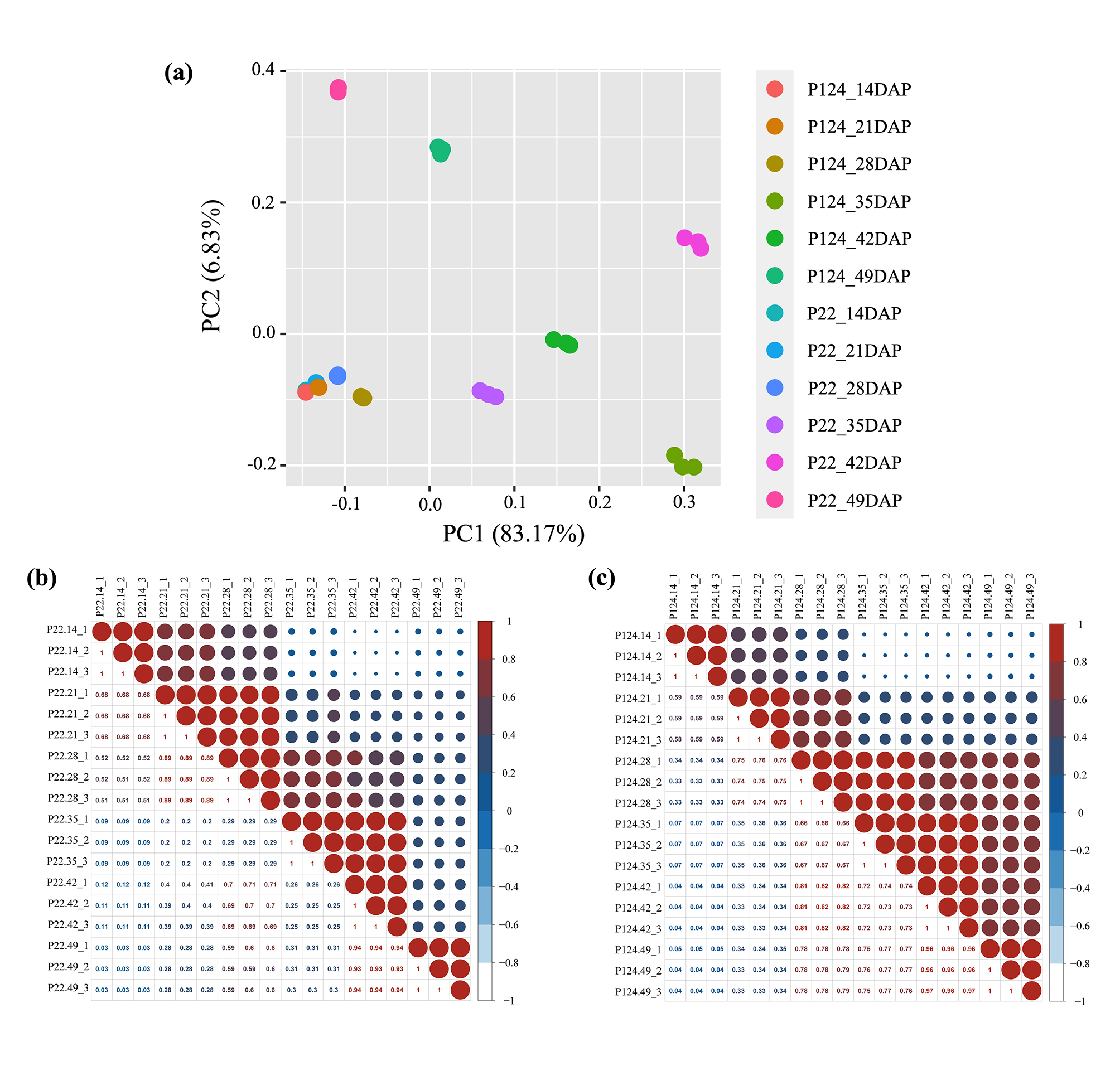

Supplement: Supplementary file 2 — Additional file 2: Fig. S2. PCA and coefficient of association analysis of both accessions (a) The 2D PCA analysis of P22 and P124, Same colors representing the biological replicates (b-c) Heatmap of all samples and correlation coefficient of P22 and P124, upper right corner representing relevance in the form of circles, and the bottom left is showing the correlation coefficient. [file 13068_2023_2275_MOESM2_ESM.tif]

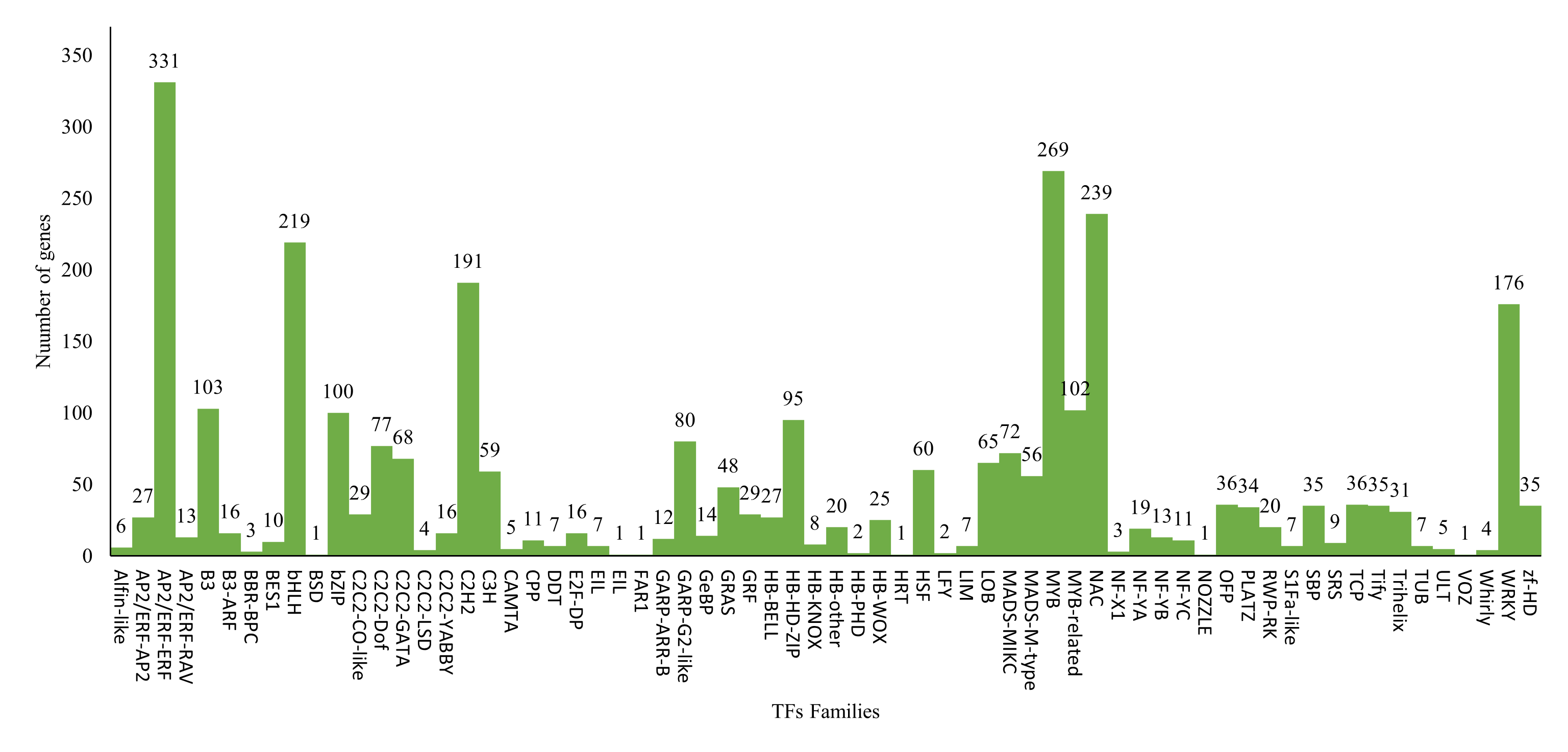

Supplement: Supplementary file 3 — Additional file 3: Fig. S3. Classification of differentially expressed TFs. [file 13068_2023_2275_MOESM3_ESM.tif]

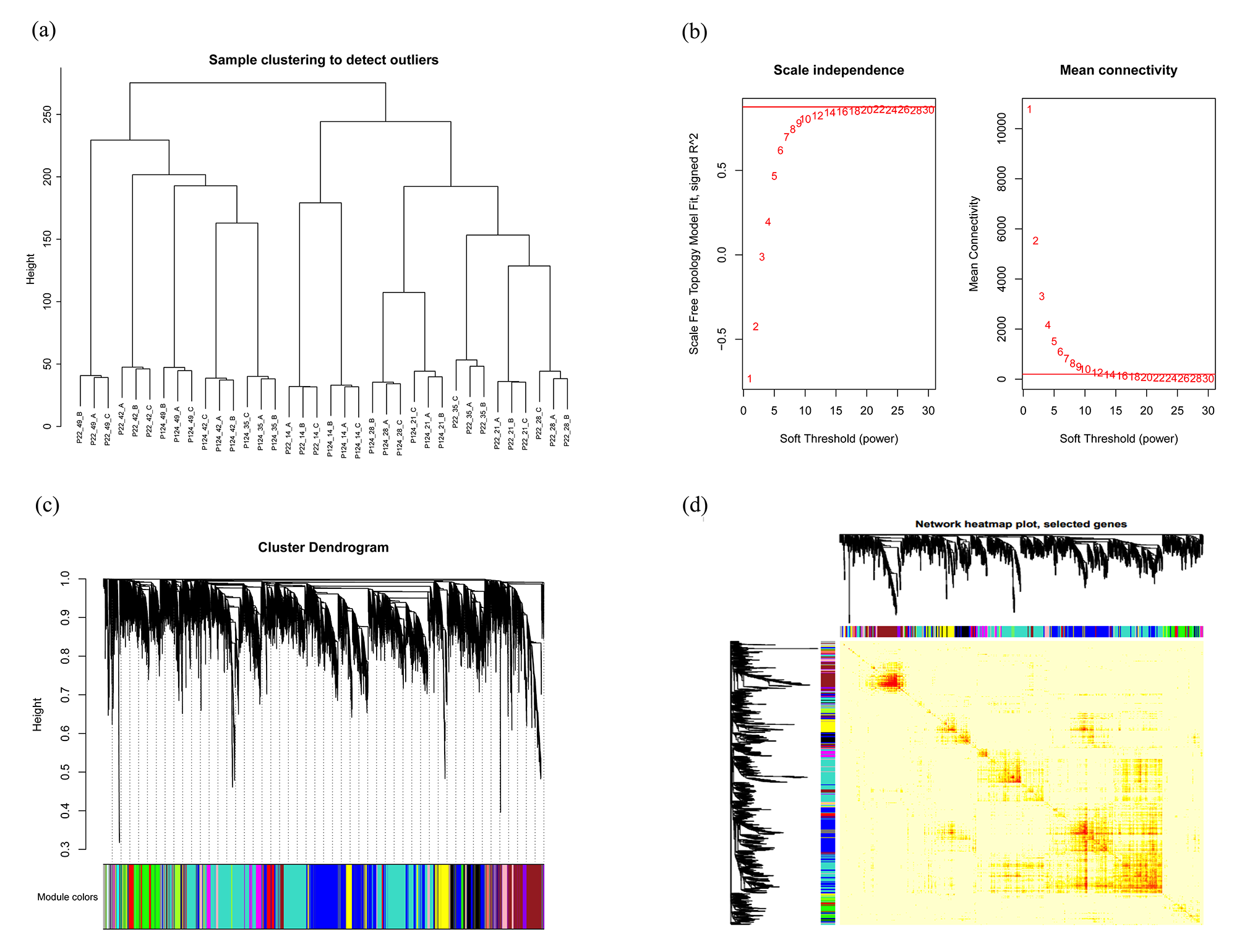

Supplement: Supplementary file 4 — Additional file 4: Fig. S4. The construction of co-expression network. (a) Clustering tree of all 36 samples; (b) Left one showing the relationship between soft threshold and scale independence. Right, one showing the relationship between soft threshold and mean connectivity; (c) The cluster dendrogram; (d) The correlation among all color modules represented in the form of heatmap, darker colors representing higher correlation. [file 13068_2023_2275_MOESM4_ESM.tif]

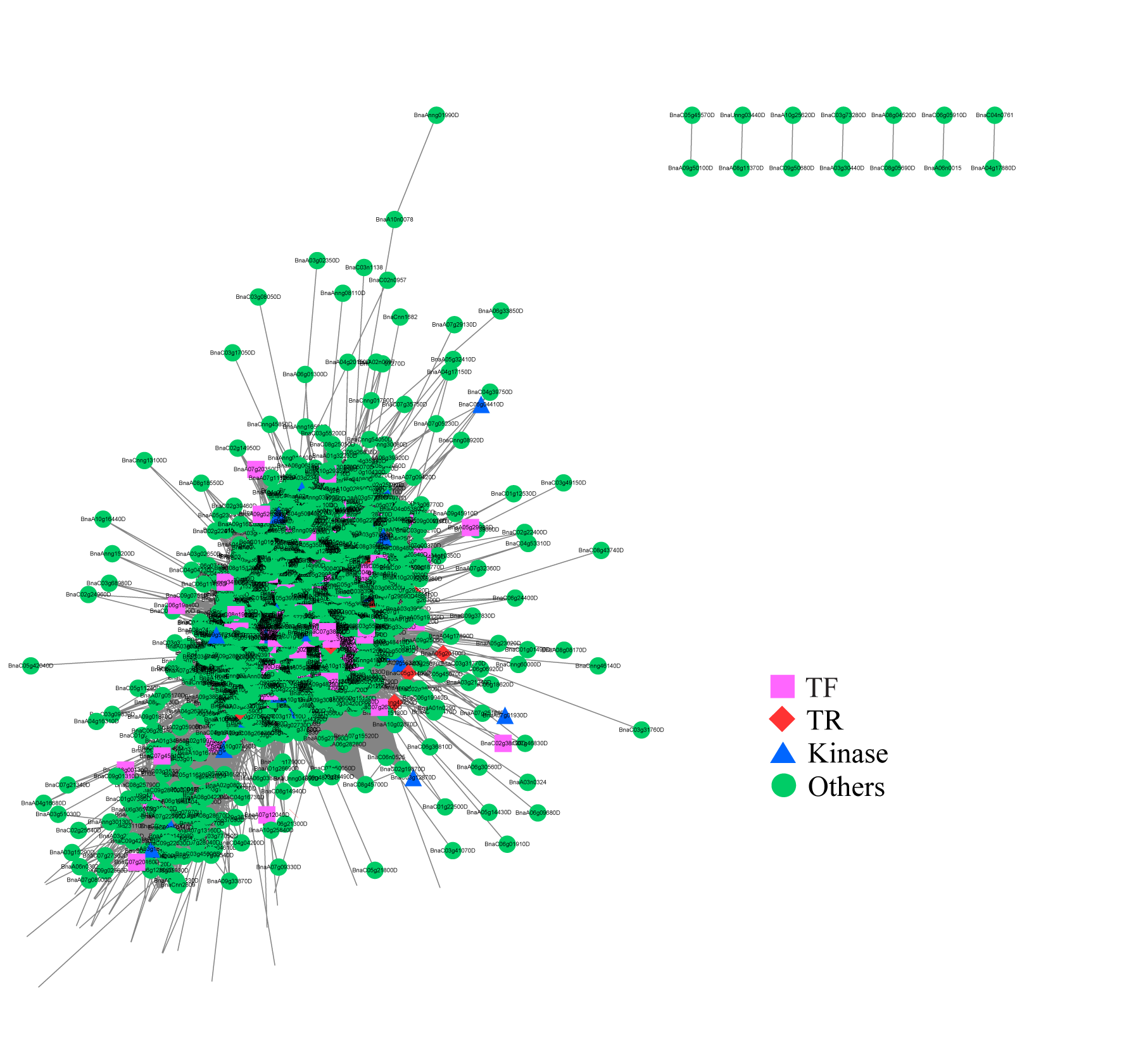

Supplement: Supplementary file 5 — Additional file 5: Fig. S5. The co-expression network of MEyellow module. Different shapes show TFs, TRs and kinases. [file 13068_2023_2275_MOESM5_ESM.tif]

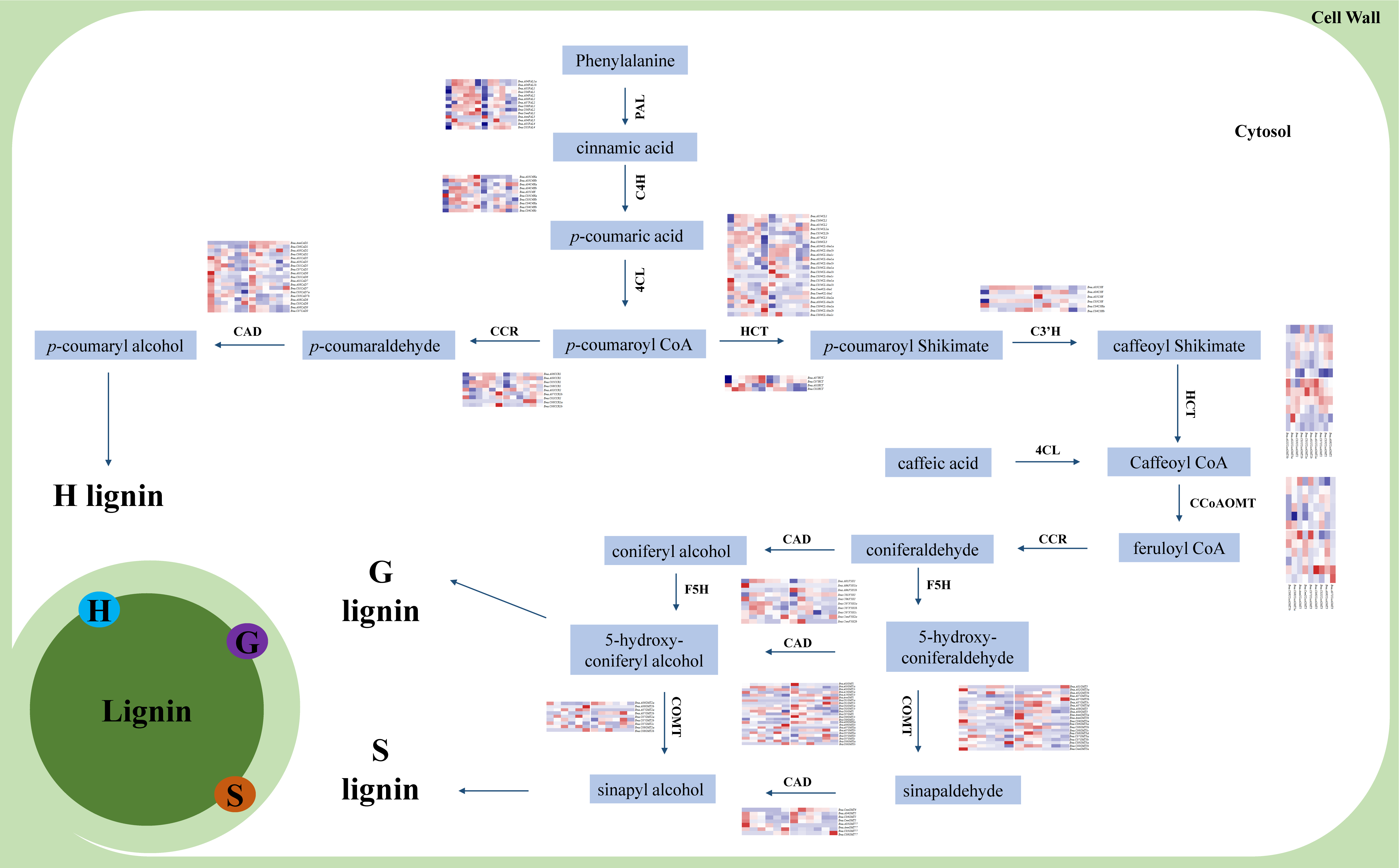

Supplement: Supplementary file 6 — Additional file 6: Fig. S6. The heatmap of lignin biosynthesis pathway. Lignin biosynthesis pathway, expression of enzymes encoded genes at each step was represented by Log2(FPKM + 1) normalized values. [file 13068_2023_2275_MOESM6_ESM.tif]

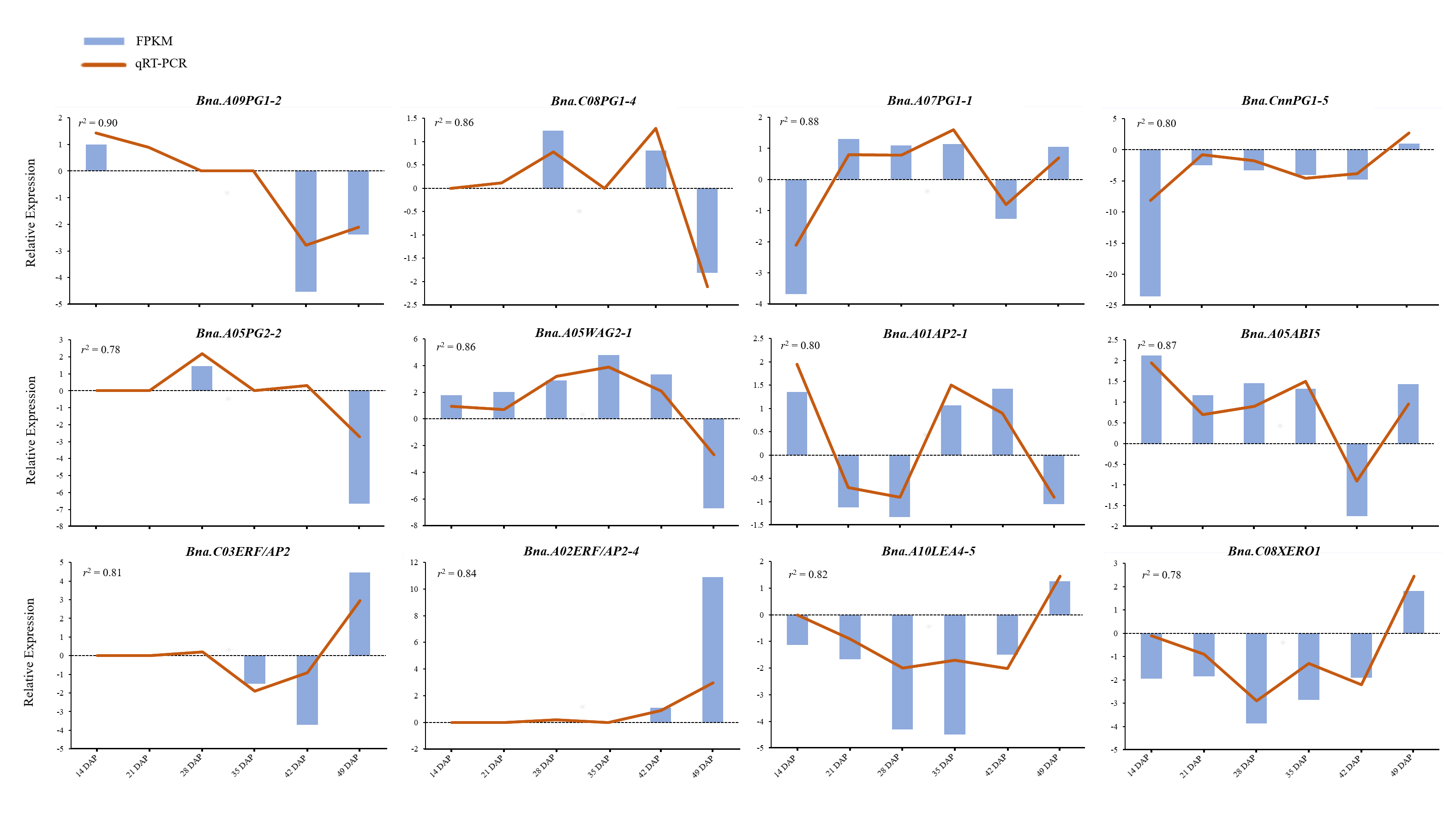

Supplement: Supplementary file 7 — Additional file 7: Fig. S7. qRT-PCR validation of DEGs. Twelve DEG genes were selected for the qRT-PCR validation. FPKM and qRT-PCR values were represented with blue and orange color, respectively. The regression (r2) coefficients were presented on the left top of each gene [file 13068_2023_2275_MOESM7_ESM.tiff]

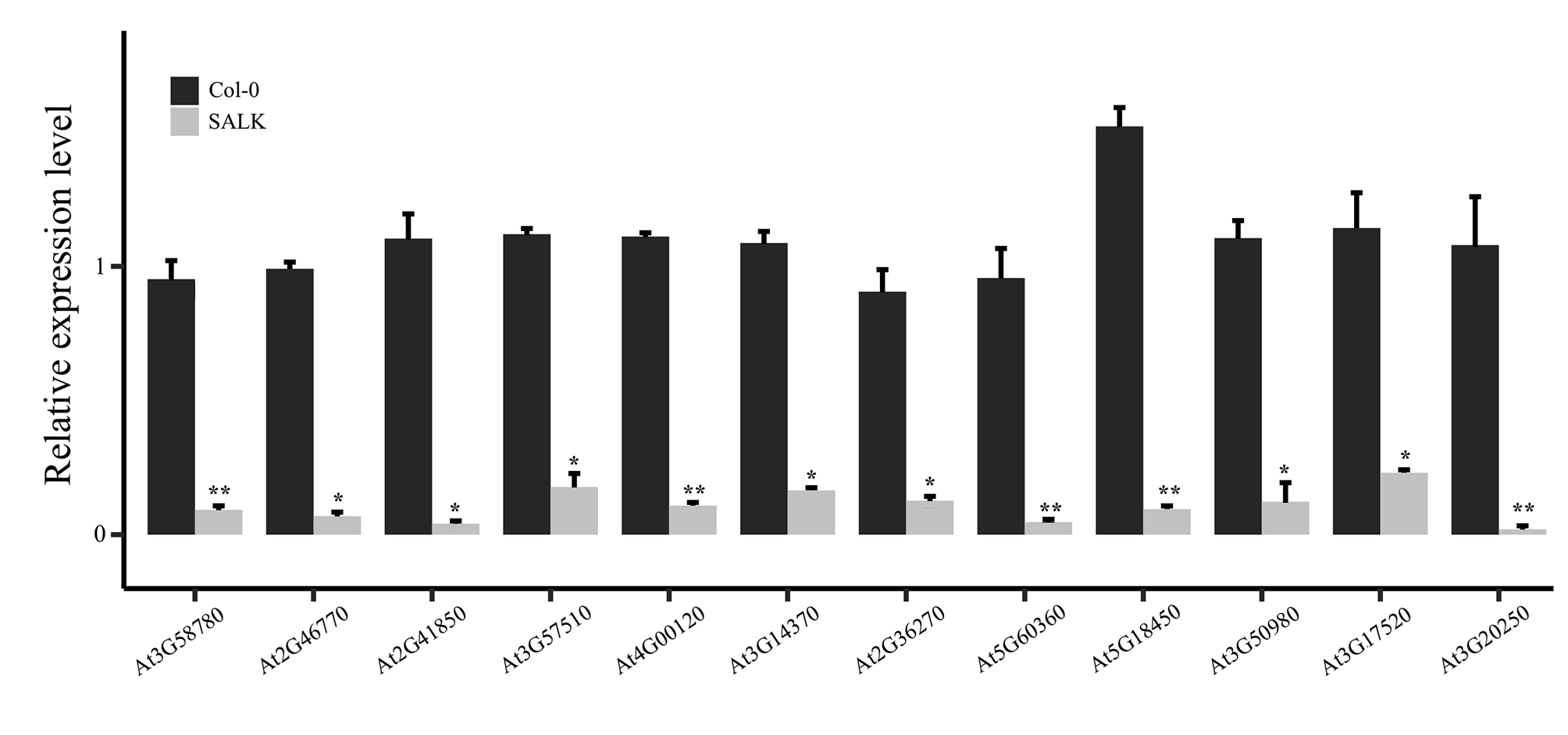

Supplement: Supplementary file 8 — Additional file 8: Fig. S8. The relative expression level WT and T-DNA mutants candidate genes. Data representing means and standard deviation (Student’s t-test; *P < 0.05, **P < 0.01). [file 13068_2023_2275_MOESM8_ESM.tif]
